# CXCR2 inhibition suppresses acute and chronic pancreatic inflammation

**DOI:** 10.1002/path.4555

**Published:** 2015-06-04

**Authors:** Colin W Steele, Saadia A Karim, Mona Foth, Loveena Rishi, Joshua DG Leach, Ross J Porter, Colin Nixon, TR Jeffry Evans, C Ross Carter, Robert JB Nibbs, Owen J Sansom, Jennifer P Morton

**Affiliations:** ^1^ Cancer Research UK Beatson InstituteGlasgowUK; ^2^ Institute of Cancer SciencesUniversity of GlasgowGlasgowUK; ^3^ Department of SurgeryGlasgow Royal InfirmaryGlasgowUK; ^4^ Centre for Immunobiology, Institute of Infection, Immunity, and InflammationUniversity of GlasgowGlasgowUK

**Keywords:** pancreatitis, CXCR2, inflammation, chemokines

## Abstract

Pancreatitis is a significant clinical problem and the lack of effective therapeutic options means that treatment is often palliative rather than curative. A deeper understanding of the pathogenesis of both acute and chronic pancreatitis is necessary to develop new therapies. Pathological changes in pancreatitis are dependent on innate immune cell recruitment to the site of initial tissue damage, and on the coordination of downstream inflammatory pathways. The chemokine receptor CXCR2 drives neutrophil recruitment during inflammation, and to investigate its role in pancreatic inflammation, we induced acute and chronic pancreatitis in wild‐type and Cxcr2^−/−^ mice. Strikingly, Cxcr2^−/−^ mice were strongly protected from tissue damage in models of acute pancreatitis, and this could be recapitulated by neutrophil depletion or by the specific deletion of Cxcr2 from myeloid cells. The pancreata of Cxcr2^−/−^ mice were also substantially protected from damage during chronic pancreatitis. Neutrophil depletion was less effective in this model, suggesting that CXCR2 on non‐neutrophils contributes to the development of chronic pancreatitis. Importantly, pharmacological inhibition of CXCR2 in wild‐type mice replicated the protection seen in Cxcr2^−/−^ mice in acute and chronic models of pancreatitis. Moreover, acute pancreatic inflammation was reversible by inhibition of CXCR2. Thus, CXCR2 is critically involved in the development of acute and chronic pancreatitis in mice, and its inhibition or loss protects against pancreatic damage. CXCR2 may therefore be a viable therapeutic target in the treatment of pancreatitis. © 2015 The Authors. The Journal of Pathology published by John Wiley & Sons Ltd on behalf of Pathological Society of Great Britain and Ireland.

## Introduction

Pancreatitis is a source of significant morbidity and mortality. However, the lack of effective interventions means the emphasis in clinical practice is on supportive care or prevention, rather than cure, and an improved understanding of the pathogenesis of pancreatic inflammation is required to develop new strategies for therapeutic intervention 1.

Acute pancreatitis arises as a result of damage to acinar cells. In humans, this is most often caused by excess alcohol intake or biliary blockade, which can be mimicked in mice by injection of the cholecystokinin analogue caerulein. Trypsinogen activation accounts for some of the pancreatic damage that develops in acute pancreatitis 2, but tissue damage is substantially augmented by the inflammatory response 3, 4. The transcription factors NF‐κB 3, AP‐1, and NFAT 5, 6 are also activated and induce the production of pro‐inflammatory mediators including CXC chemokines (eg CXCL1 7 and CXCL2 8), CC chemokines (including CCL2 9), and cytokines such as interleukin (IL)‐6 10, TNFα 11, and IL‐1β. These mediators drive an acute inflammatory response characterized by the influx of innate immune cells, first neutrophils and then monocytes, to the site of damage. Deletion of IL‐1β and TNF reduces the severity of acute pancreatitis 11, while TLR4 deletion reduces innate immune cell recruitment to the pancreas, possibly by suppressing CXCL2 production 12. The infiltrating leukocytes augment acinar cell damage and intra‐acinar cell trypsinogen activation 13, and the release of reactive oxygen species or proteases may have a key role in these processes 13, 14, 15. In most cases, acute pancreatitis resolves with tissue repair and regeneration, but in severe cases a cycle of damage and inflammation is set in motion whereby pro‐inflammatory cytokines act systemically and, unless reversed, result in systemic inflammatory response syndrome and potentially organ failure and death.

The pathogenesis of chronic pancreatitis is less well understood 5. Current thinking asserts that it is a ‘two‐hit’ phenomenon in which initial damage generates acute pancreatitis and a second ‘hit’ drives progression to chronic pancreatitis. This second hit reprogrammes the acute inflammatory response to promote chronic inflammation, stellate cell activation, and fibrosis, thus instigating a necrosis/fibrosis cycle that is perpetuated by recurrent acute attacks 16. Effective therapies must therefore allow healing and regeneration after acute pancreatitis and before fibrosis and chronic pancreatitis develop.

Due to their key role in inflammation, neutrophils are considered a potential therapeutic target in acute pancreatitis. The chemokine receptor CXCR2 is known to make a major contribution to neutrophil migration *in vivo*. In humans, CXCR2 acts as a receptor for seven different chemokines (CXCL1–3 and CXCL5–8), two of which (CXCL6 and CXCL8) also signal through the closely related receptor CXCR1 17. Mice lack CXCL8 (previously known as IL‐8) and only a single orthologue exists for CXCL5 and CXCL6, so CXCR2 only has five chemokine ligands in mice, although non‐chemokine ligands have also been reported to activate this receptor 18, 19. Whilst CXCR2 has been implicated in a diverse range of cellular processes, such as development 20, angiogenesis 21, senescence 22, and tumour cell proliferation and invasion 23, it is best known for its ability to control leukocyte migration. It plays a particularly prominent role in directing neutrophil migration, including release from the bone marrow and recruitment into inflamed tissues 24, 25. However, functional CXCR2 expression has also been reported on other leukocytes, including T‐lymphocytes, NK cells, and monocytes in humans 26, 27, 28, 29, and monocytes in mice 30, 31, 32.

Here, using genetically modified mice, we show that CXCR2 is critical for the recruitment of neutrophils into the pancreas in models of acute and chronic pancreatitis, and that this plays a key role in driving the acinar cell damage seen in these models. Moreover, a CXCR2 inhibiting peptide, ‘pepducin’, protects wild‐type mice against acute and chronic pancreatitis and, significantly, can reverse established pancreatic inflammation. Thus, inhibitors of CXCR2 signalling could be of benefit in the treatment of both acute and chronic pancreatitis in humans.

## Materials and methods

### Mice

Animals of BALB/c (all *Cxcr2^−/−^* and controls) or C57Bl/6 (all *Cxcr2^fl/fl^* mice and controls) background were maintained in conventional animal facilities and monitored daily. Experiments were carried out in compliance with UK Home Office guidelines under licence and approved by the local ethical review committee. Wild‐type animals were purchased from Charles River Laboratories (Margate, Kent, UK). *Cxcr2^−/−^* mice were obtained from Jackson Laboratories (Maine, USA), and genotyped by Transnetyx (Cordova, TN, USA). As *Cxcr2^−/−^* mice can be smaller than average, we used only mice of a comparable size to controls for all experiments.

### Experimental pancreatitis

Acute pancreatitis was induced by intraperitoneal (i.p.) injection of 0.2 mg/kg caerulein (Sigma Aldrich, St Louis, MO, USA) every hour for 6 h. Animals were sacrificed 1 or 18 h after the final injection. Chronic pancreatic inflammation was induced by i.p. injection of 0.2 mg/kg caerulein once daily for a continuing cycle of 5 days on, 2 days off. Animals were aged to 6 weeks or 9 months. Groups of five age‐matched wild‐type and *Cxcr2^−/−^* mice were used.

### Treatment studies

Healthy, age‐matched mice were randomly assigned to control or treatment in each case and treated and assessed at the same time. Further details may be found in the Supplementary materials and methods.

### Assessment of circulating cells

Blood was obtained *post mortem* from mice by cardiac puncture, collected into EDTA‐coated tubes, and analysed immediately using an ADVIA2120 Haematology system (Siemens, Munich, Germany) by the University of Glasgow Veterinary Diagnostics Service.

### Human pancreatic tissue

Tissue from pancreata resected from human patients with pancreatitis was obtained from Glasgow Biorepository. Expression was assessed by immunohistochemistry.

### Immunohistochemistry

Sections were stained using standard immunohistochemistry protocols. Further details may be found in the Supplementary materials and methods.

### Flow cytometry

Flow cytometry was performed using standard protocols. Further details may be found in the Supplementary materials and methods.

## Results

### CXCR2 drives acute pancreatic inflammation and acinar damage

**Figure 1:**
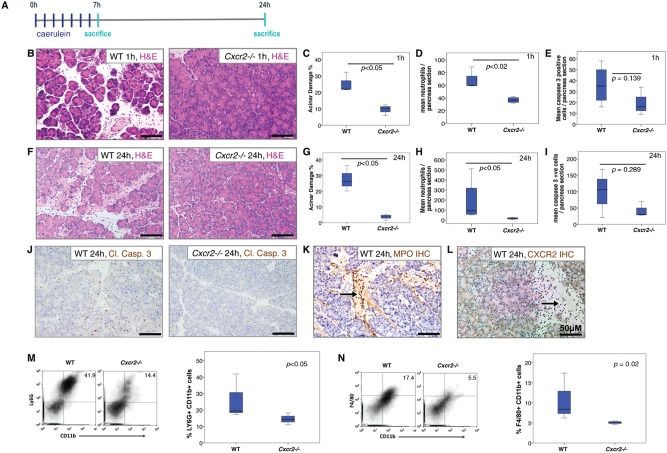
Cxcr2 deletion protects the pancreas from the effects of acute pancreatitis. (A) Schematic diagram of induction of acute pancreatitis, showing the frequency of caerulein injections and sampling time‐points. (B) H&E staining of pancreata from WT (left) and Cxcr2^−/−^ (right) mice 1 h after induction of acute pancreatitis. (C–E) Boxplots showing quantification of (C) acinar damage (p < 0.05, Mann–Whitney, n = 4), (D) MPO^+^ neutrophils (p < 0.02, Mann–Whitney, n = 4), and (E) cleaved caspase 3 (p = 0.139, Mann–Whitney, n = 4) in WT and Cxcr2^−/−^ mice 1 h after induction of acute pancreatitis. (F) H&E staining of pancreata from WT (left) and Cxcr2^−/−^ (right) mice 24 h after induction of acute pancreatitis. (G–I) Boxplots showing quantification of (G) acinar damage (p < 0.05, Mann–Whitney, n = 4), (H) MPO^+^ neutrophils (p < 0.05, Mann–Whitney, n = 4 mice), and (I) cleaved caspase 3 (p = 0.289, Mann–Whitney, n = 4) in WT and Cxcr2^−/−^ mice 24 h after induction of acute pancreatitis. (J) IHC for cleaved caspase 3 in pancreata from WT (left) and Cxcr2^−/−^ (right) mice 24 h after induction of acute pancreatitis. (K) IHC for MPO to detect neutrophils infiltrating the pancreas 24 h after induction of acute pancreatitis. (L) CXCR2 staining 24 h after induction of acute pancreatitis. Arrows indicate positive cells. (M, N) FACS analysis confirms the decrease in recruitment of (M) CD11b^+^ Ly6G^+^ neutrophils (quantified on right, n ≥ 3) and (N) CD11b^+^ F4/80^+^ monocytes (quantified on right, n ≥ 3) to the pancreas of Cxcr2^−/−^ mice 24 h after induction of acute pancreatitis.

To assess the role of CXCR2 in the pathogenesis of acute pancreatitis, we compared the responses of *Cxcr2^−/−^* mice 24 and age‐matched wild‐type mice to repeated treatment with caerulein 33. A series of seven hourly injections of caerulein results in a condition that mimics the pathology of human acute pancreatitis 34 (Figure 1A).

Mice were sacrificed 1 h after the final injection of caerulein, after the first peak of trypsin activity in this model 35, 36. From inspection of H&E‐stained sections, it was clear that acute pancreatic damage had been induced by the caerulein in wild‐type and *Cxcr2^−/−^* pancreata, and apoptotic figures and inflammatory cells were evident (Figure 1B). Acinar damage was assessed using a standard scoring system based on morphometry 2 (Figure 1C). Immunohistochemistry (IHC) showed that the immune cell infiltrate at this time‐point consisted predominantly of myeloperoxidase (MPO)‐positive neutrophils, and these cells were substantially reduced in *Cxcr2^−/−^* pancreata (Figure 1D). Apoptotic cells were quantified by IHC for cleaved caspase 3, but no significant difference was observed between groups (Figure 1E), suggesting that the damage sustained from caerulein‐induced pancreatic enzyme stimulation was not substantially affected by CXCR2 deficiency. Interestingly, despite containing similar numbers of apoptotic cells, the pancreata of *Cxcr2^−/−^* mice exhibited significantly reduced levels of acinar damage compared with wild‐type mice.

To determine the contribution of CXCR2 to secondary inflammation and damage in acute pancreatitis, wild‐type and *Cxcr2^−/−^* mice were administered seven hourly injections of caerulein and sacrificed 24 h after the first injection (Figure 1A). At this time, inflammatory infiltrates, oedema, and necrosis are present in the pancreas 34. Wild‐type pancreata were visibly oedematous compared with *Cxcr2^−/−^* pancreata. H&E staining revealed tissue oedema and leukocyte infiltrates in wild‐type pancreata that were not seen in their *Cxcr2^−/−^* counterparts (Figure 1F). When acinar damage was quantified, it was found to be approximately five‐fold higher in the pancreata of wild‐type mice than in *Cxcr2^−/−^* animals (Figure 1G). Similar to the 1 h time‐point, no significant difference was observed in the number of apoptotic cells between groups (Figure 1I), as quantified by IHC for cleaved caspase 3 (Figure 1J). IHC confirmed that the immune cell infiltrate consisted predominantly of MPO‐positive neutrophils at this time‐point (Figure 1K), and these cells were substantially reduced in *Cxcr2^−/−^* pancreata (Figure 1H). As expected, cells within the infiltrate of wild‐type pancreata expressed CXCR2 (Figure 1L). Presence of macrophages was assessed at both 1‐h and 24‐h time‐points in wild‐type mice, and while very few macrophages were seen at 1 h after pancreatitis induction, high numbers were seen at the 24‐h time‐point (Supplementary Figure 1A). Very few CD3‐positive T cells were present in the pancreata of either wild‐type or *Cxcr2^−/−^* mice (Supplementary Figure 1B). We also analysed cells isolated from the pancreata of wild‐type and *Cxcr2^−/−^* mice by flow cytometry and found that in keeping with our immunohistochemical data, the numbers of CD11b^+^Ly6G^+^ and CD11b^+^F4/80^+^ cells were reduced in pancreata following pancreatitis induction (Figures 1M and 1N and Supplementary Figure 2).

Collectively, these data show that CXCR2 deficiency prevents leukocyte recruitment into the pancreas and protects the pancreatic parenchyma from inflammation‐induced damage. Importantly, this reduction in recruitment into the pancreas is not due to a lack of circulating neutrophils in *Cxcr2^−/−^* mice, as shown by full blood counts from these mice (Supplementary Figure 3). Interestingly, although circulating leukocyte levels are higher in *Cxcr2^−/−^* mice, due to failed homing of the neutrophils, under inflammatory conditions this appears to be corrected.

### Deletion of Cxcr2 from myeloid cells is sufficient for protection from acute pancreatitis

**Figure 2:**
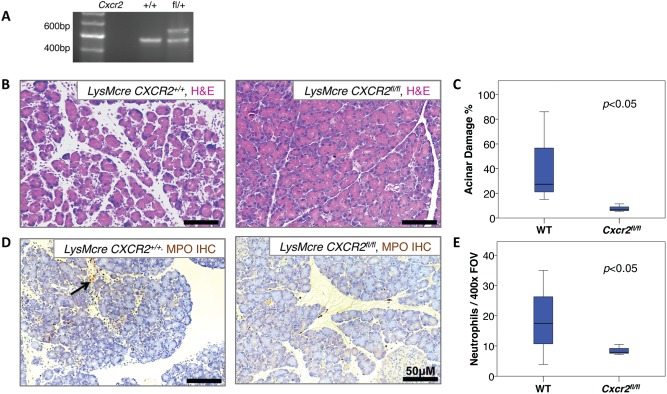
CXCR2 expression on myeloid cells determines damage in acute pancreatitis. (A) PCR of DNA from wild‐type mice and mice heterozygous for the Cxcr2^fl/fl^ allele. (B) H&E staining demonstrating significant pancreatic damage in LysMCre, Cxcr2^+/+^ mice (left) compared with LysMCre, Cxcr2^fl/fl^ mice (right), 24 h following induction of acute pancreatitis. (C) Boxplot showing quantification of acinar damage as a percentage of total acinar tissue per × 200 field of view. p < 0.05, Mann–Whitney, n = 3 mice. (D) Immunohistochemistry for MPO, demonstrating MPO^+^ neutrophil infiltrate in LysMCre, Cxcr2^+/+^ mice (left) compared with LysMCre, Cxcr2^fl/fl^ mice (right), 24 h following induction of acute pancreatitis. (E) Boxplot showing quantification of MPO^+^ neutrophils. Note the significantly fewer MPO^+^ neutrophils within the pancreas of LysMCre, Cxcr2^fl/fl^ mice compared with LysMCre, Cxcr2^+l+^ mice. Black arrow denotes neutrophil infiltrate. p <0.05, Mann–Whitney, n = 3 mice.

In inflamed wild‐type pancreata, CXCR2 expression is restricted to infiltrating leukocytes (Figure 1L). In order to determine whether the protection offered by *Cxcr2* deletion was a result of loss of CXCR2 from myeloid cells, we developed a conditional floxed allele of *Cxcr2* (Figure 2A). To specifically delete CXCR2 in myeloid cells, we crossed these mice with animals expressing *Cre* recombinase under the control of the lysozyme M promoter (*LysMCre*) 37. Acute pancreatitis was induced in cohorts of *LysMCre, Cxcr2^+/+^* and *LysMCre, Cxcr2^fl/fl^* mice, and as above, animals were analysed 24 h after the first caerulein injection. We observed a striking level of pancreatic parenchymal damage in *LysMCre*, *Cxcr2^+/+^* mice as expected. In contrast, *LysMCre, Cxcr2^fl/fl^* mice were completely protected (Figures 2B and 2C). In addition, MPO^+^ neutrophils were virtually absent from the pancreata of *LysMCre, Cxcr2^fl/fl^* mice compared with *LysMCre, Cxcr2^+/+^* controls (Figures 2D and 2E). Importantly, *Pdx1‐Cre, Cxcr2^fl/fl^* mice, in which *Cxcr2* is deleted from pancreatic epithelial cells, were not protected from pancreatitis (Supplementary Figure 4). Thus, CXCR2 deficiency in myeloid cells interferes with neutrophil recruitment to the pancreas and protects the pancreas from inflammation.

### Neutrophil depletion and CXCR2 inhibition protect against acute pancreatitis

Neutrophils are by far the most dominant source of CXCR2 in mice, and the phenotypes in *Cxcr2^−/−^* mice are likely to be due to loss of CXCR2 from neutrophils. The results predict that neutrophil depletion or the pharmacological targeting of CXCR2 should protect wild‐type mice from acinar cell damage and pancreatic inflammation. To examine this, we used anti‐Ly6G monoclonal antibody (1A8) to deplete Ly6G^+^ neutrophils and a CXCR2 targeting peptide ‘pepducin’ that interferes with the intracellular coupling of the G‐protein loops of CXCR2 to prevent CXCR2 signalling 19.

**Figure 3:**
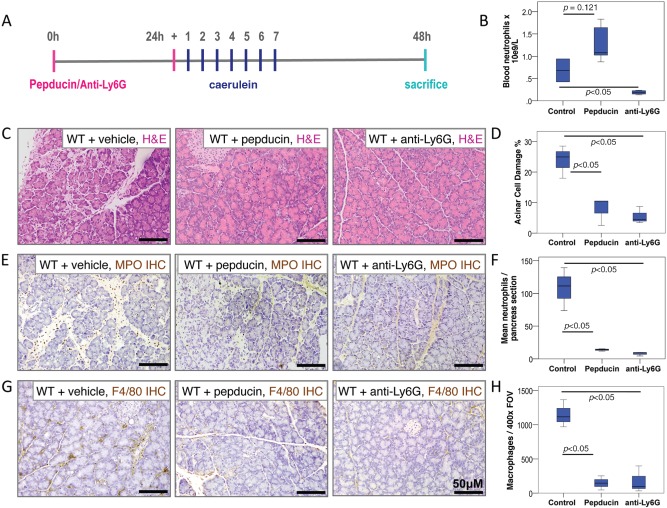
CXCR2 inhibition protects the pancreas from the effects of acute pancreatitis. (A) Schematic diagram of induction of acute pancreatitis, showing timing of treatment, frequency of caerulein injections, and sampling time‐points. (B) Boxplot showing quantification of circulating blood neutrophils compared between treatment groups. Control versus CXCR2 pepducin, p = 0.121. Control versus anti‐Ly6G, p < 0.05, Mann–Whitney n = 5 mice in each group. (C) H&E staining of pancreata from vehicle‐treated (left), CXCR2 pepducin‐treated (middle), and anti‐Ly6G‐treated (right) wild‐type mice 24 h following induction of acute pancreatitis. (D) Boxplot showing quantification of acinar damage compared between treatment groups. Control versus CXCR2 pepducin, p < 0.05. Control versus anti‐Ly6G, p < 0.05, Mann–Whitney, n = 5 mice in each group. (E) Immunohistochemistry for MPO to detect neutrophils in pancreata from vehicle‐treated (left), CXCR2 pepducin‐treated (middle), and anti‐Ly6G‐treated (right) wild‐type mice 24 h after induction of acute pancreatitis. (F) Boxplot showing quantification of MPO^+^ neutrophils compared between treatment groups. Control versus CXCR2 pepducin, p < 0.05. Control versus anti‐Ly6G, p < 0.05, Mann–Whitney, n = 5 mice in each group. (G) Immunohistochemistry for F4/80 to detect macrophages in pancreata from vehicle‐treated (left), CXCR2 pepducin‐treated (middle), and anti‐Ly6G‐treated (right) wild‐type mice 24 h after induction of acute pancreatitis. (H) Boxplot showing quantification of F4/80^+^ macrophages compared between treatment groups. Control versus CXCR2 pepducin, p < 0.05. Control versus anti‐Ly6G, p < 0.05, Mann–Whitney, n = 5 mice in each group.

First, we assessed whether prophylactic treatment with pepducin or anti‐Ly6G antibody could protect against acute pancreatitis. Wild‐type mice were dosed with pepducin, anti‐Ly6G antibody or control 24 h and 1 h before inducing acute pancreatitis (Figure 3A). Animals were sacrificed 24 h after induction of acute pancreatitis. We confirmed that anti‐Ly6G reduced the circulating neutrophil counts significantly compared with control mice (Figure 3B). While there was no significant difference between control and CXCR2 pepducin‐treated animals, there was a trend towards higher numbers of circulating neutrophils in CXCR2 pepducin‐treated mice, perhaps due to reduced homing to the pancreas. As expected, pancreata from control mice showed evidence of tissue damage, apoptotic bodies within acinar cells, and infiltration by inflammatory cells (Figures 3C, 3E, and 3G). In contrast, the pancreata of wild‐type mice treated with pepducin or anti‐Ly6G showed a significant reduction in acinar cell damage (Figures 3C and 3D) and were virtually devoid of MPO^+^ neutrophils (Figures 3E and 3F). As with CXCR2 deficiency, the treatment of wild‐type mice with pepducin or anti‐Ly6G resulted in a substantial reduction in the number of F4/80^+^ macrophages present in the pancreas 24 h after the first caerulein injection (Figures 3G and 3H).

Taken together, the data suggest that inhibition of neutrophil infiltration to the pancreas, either by *Cxcr2* deletion, CXCR2 inhibition or neutrophil depletion, restricts pancreatic damage in response to caerulein‐induced acute pancreatitis and prevents the development of a pancreatic monocyte–macrophage infiltrate.

### CXCR2 inhibition ameliorates tissue damage in mice with ongoing acute pancreatitis

**Figure 4:**
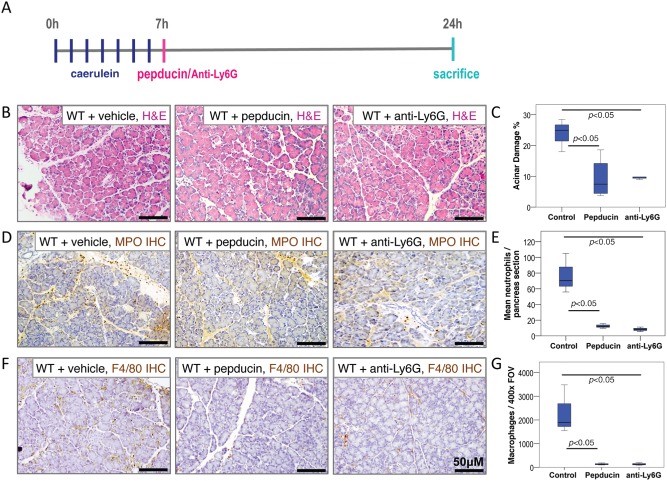
CXCR2 inhibition following induction of acute pancreatitis rescues the pancreatic parenchymal damage phenotype in a neutrophil‐dependent manner. (A) Schematic diagram of induction of acute pancreatitis, showing timing of treatment, frequency of caerulein injections, and sampling time‐points. (B) H&E staining of pancreata from vehicle‐treated (left), CXCR2 pepducin‐treated (middle), and anti‐Ly6G‐treated (right) wild‐type mice 24 h following induction of acute pancreatitis. (C) Boxplot showing quantification of acinar damage compared between treatment groups. Control versus CXCR2 pepducin, p < 0.05. Control versus anti‐Ly6G, p < 0.05, Mann–Whitney, n = 5 mice in each group. (D) Immunohistochemistry for MPO to detect neutrophils in pancreata from vehicle‐treated (left), CXCR2 pepducin‐treated (middle), and anti‐Ly6G‐treated (right) wild‐type mice 24 h after induction of acute pancreatitis. (E) Boxplot showing quantification of MPO^+^ neutrophils compared between treatment groups. Control versus CXCR2 pepducin, p < 0.05. Control versus anti‐Ly6G, p < 0.05, Mann–Whitney, n = 5 mice in each group. (F) Immunohistochemistry for F4/80 to detect macrophages in pancreata from vehicle‐treated (left), CXCR2 pepducin‐treated (middle), and anti‐Ly6G‐treated (right) wild‐type mice 24 h after induction of acute pancreatitis. (G) Boxplot showing quantification of F4/80^+^ macrophages compared between treatment groups. Control versus CXCR2 pepducin, p < 0.05. Control versus anti‐Ly6G, p < 0.05, Mann–Whitney, n = 5 mice in each group.

Next, in order to assess the potential therapeutic utility of CXCR2 inhibition in acute pancreatitis, we investigated whether inhibition of CXCR2 or neutrophil ablation could reverse or dampen ongoing pancreatic inflammation. As before, we induced acute pancreatitis in wild‐type mice by seven hourly injections of caerulein, but this time we treated with CXCR2 pepducin, anti‐Ly6G antibody or control 1 h after the final injection (Figure 4A). At this time in this model, the trypsin activity has already peaked 2 and substantial chemical and inflammation‐induced damage is apparent in the pancreas (Figure 1). Animals were sacrificed 24 h after pepducin or anti‐Ly6G treatment. H&E staining revealed greater immune cell infiltrate and tissue damage in control animals compared with CXCR2 pepducin‐treated or anti‐Ly6G antibody‐treated mice (Figure 4B). Upon quantitation, it was clear that acinar damage was not reduced to the low levels observed in the prevention experiment (Figure 3), but, nonetheless, treatment with pepducin or anti‐Ly6G was able to prevent tissue damage caused by ongoing acute pancreatitis (Figure 4C). As in previous experiments, both CXCR2 inhibition and neutrophil ablation in this context significantly reduced the number of neutrophils (Figures 4D and 4E) and macrophages (Figures 4F and 4G) in the pancreata.

Importantly, these data show that blocking CXCR2 or depleting neutrophils can significantly reduce pancreatic damage, even when it is initiated after the onset of acute pancreatitis. CXCR2 may therefore have considerable potential as a therapeutic target in the treatment of acute pancreatitis.

### CXCR2 deletion protects against the effects of recurrent acute pancreatic damage

**Figure 5:**
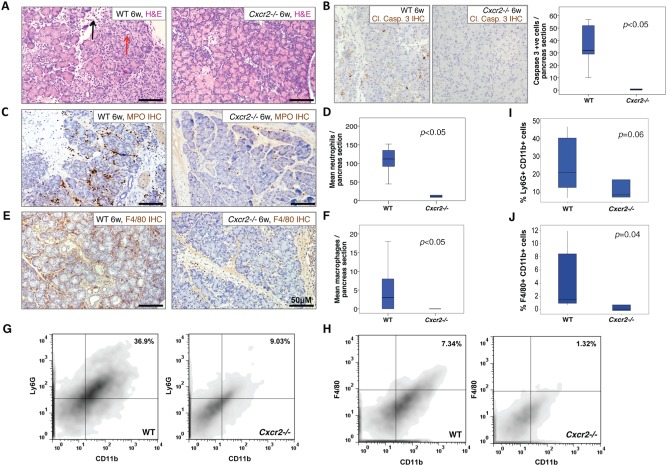
Cxcr2 deletion protects against the effects of recurrent acute pancreatic inflammation. (A) H&E staining of pancreata from wild‐type (left) and Cxcr2^−/−^ (right) mice following 6 weeks of pancreatic inflammation. Red arrow denotes necrotic cell; black arrow significant immune cell infiltrate. (B) Immunohistochemistry for cleaved caspase 3 to detect apoptotic cells in the pancreas of wild‐type (left) and Cxcr2^−/−^ (middle) mice following 6 weeks of pancreatic inflammation. Right panel: boxplot showing quantification of cleaved caspase 3 staining. p < 0.05, Mann–Whitney, n = 4 mice. (C) Immunohistochemistry for MPO to detect neutrophils infiltrating the pancreas of wild‐type (left) and Cxcr2^−/−^ (right) mice following 6 weeks of pancreatic inflammation. (D) Boxplot showing quantification of MPO‐positive neutrophils in the pancreas of wild‐type and Cxcr2^−/−^ mice following 6 weeks of pancreatic inflammation. p < 0.05, Mann–Whitney, n = 3 mice. (E) Immunohistochemistry for F4/80 to detect macrophages in the pancreas of wild‐type (left) and Cxcr2^−/−^ (right) mice following 6 weeks of pancreatic inflammation. (F) Boxplot showing quantification of F4/80‐positive macrophages in the pancreas of wild‐type and Cxcr2^−/−^mice following 6 weeks of pancreatic inflammation. p < 0.05, Mann–Whitney, n = 3 mice. (G–J) FACS analysis confirms the decrease in infiltration of (G) CD11b^+^ Ly6G^+^ neutrophils (quantified in I, n ≥ 3) and (H) CD11b^+^ F4/80^+^ macrophages (quantified in J, n ≥ 3) to the pancreas of Cxcr2^−/−^ mice following 6 weeks of pancreatic inflammation.

Chronic pancreatitis is a multifactorial disease that often arises in response to recurrent bouts of acute, often subclinical, pancreatitis. Recurrent inflammation generates a cycle of leukocyte infiltration (particularly neutrophils and monocytes), stellate cell activation [by macrophage‐derived cytokines including platelet‐derived growth factor and transforming growth factor beta (TGFβ)], and fibrotic tissue deposition. To investigate whether CXCR2 might play a role in this process, we wanted to examine the role of CXCR2 in a mouse model of chronic pancreatitis. Chronic pancreatitis is most often induced over a period of 6 weeks via recurrent acute pancreatitis twice weekly 38. However, this model represents a significant necrotic insult to the pancreas and relies on extensive damage and repair. We therefore decided to generate chronic pancreatitis in mice using a low‐grade regimen of recurrent intra‐peritoneal caerulein injections five days per week for 6 weeks 39. When sacrificed at the 6‐week time‐point, wild‐type pancreata had significant inflammatory infiltrate and tissue damage (Figure 5A, left), but no evidence of fibrosis. In contrast, the pancreata of *Cxcr2* ^−/−^ mice were almost devoid of inflammatory cells (Figure 5A, right) and virtually no apoptotic cells were present (Figure 5B). MPO^+^ cells and F4/80^+^ macrophages were present in wild‐type pancreata but were barely detectable in *Cxcr2* ^−/−^ mice (Figures 5C, 5D and 5F, 5G). These data were confirmed by flow cytometry (Figures 5E and 5H–5J). Full blood counts again showed that the lack of neutrophils recruited to the pancreata of *Cxcr2* ^−/−^ mice is not due to low numbers of circulating cells (Supplementary Figure 5). Thus, *Cxcr2* ^−/−^ mice appeared to be completely protected from the effects of low‐grade pancreatic inflammation.

In keeping with this hypothesis, when we stained pancreata from wild‐type and *Cxcr2* ^−/−^ mice 24 h following induction of acute pancreatitis, we found occasional cells positive for nuclear NF‐κB‐p65, indicative of NF‐κB signalling, in wild‐type, but not in *Cxcr2* ^−/−^ pancreata. After 6 weeks of chronic inflammation, the number of cells positive for nuclear NF‐κB increased in wild‐type pancreata; however, we only observed cytoplasmic staining in *Cxcr2* ^−/−^ mice (Supplementary Figure 6).

### Cxcr2 deletion protects against many features of chronic pancreatitis

**Figure 6:**
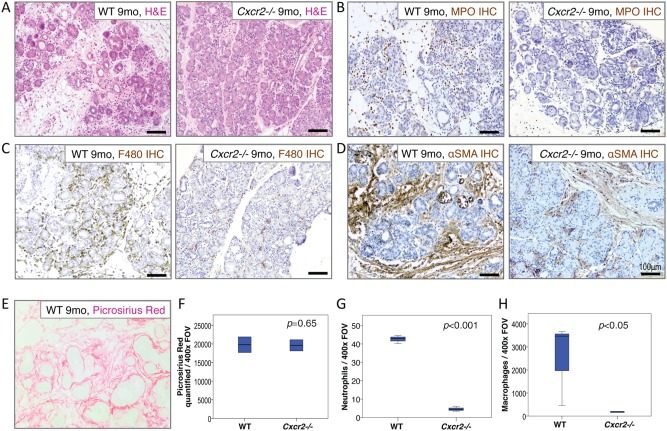
Chronic pancreatitic changes are mediated by neutrophils and macrophages and are ameliorated by Cxcr2 deletion. (A) H&E staining demonstrating parenchymal changes after 9 months of pancreatic inflammation in wild‐type (left) and Cxcr2^−/−^mice (right). (B) MPO immunohistochemistry confirms significant numbers of neutrophils infiltrating the pancreas of wild‐type mice (left) compared with Cxcr2^−/−^ mice (right). (C) F4/80 immunohistochemistry confirms significant numbers of macrophages in the pancreas of wild‐type mice (left) compared with Cxcr2^−/−^ mice (right). (D) αSMA immunohistochemistry demonstrates stellate cell activation after 9 months of pancreatic inflammation in wild‐type (left) and Cxcr2^−/−^ mice (right). (E) Fibrosis in wild‐type pancreata demonstrated by picrosirius red staining for collagen I. (F) Boxplot showing quantification of fibrosis in the pancreata of wild‐type and Cxcr2^−/−^ mice. p = 0.65, Mann–Whitney. (G) Boxplot showing quantification of MPO^+^ neutrophils in the pancreata of wild‐type mice compared with Cxcr2^−/−^ mice. p < 0.001, Mann–Whitney. (H) Boxplot showing quantification of macrophages in the pancreata of wild‐type mice compared with Cxcr2^−/−^mice. p < 0.05, Mann–Whitney, n = 3 mice in each group.

To model chronic pancreatitis more accurately, we extended our regime and injected mice i.p. with caerulein for 9 months, 5 days per week (Figure 6). This was expected to induce significant fibrosis and tissue atrophy in the pancreas 39. In wild‐type mice, there was significant leukocyte infiltration of the pancreas with resultant tissue damage and extensive atrophy. In contrast, *Cxcr2* ^−/−^ pancreata had fewer areas of tissue atrophy and little evidence of leukocyte infiltrate. In wild‐type mice, the inflammatory infiltrate consisted mainly of MPO^+^ neutrophils and F4/80^+^ macrophages (Figures 6B, 6C and 6G, 6H).

We hypothesized that the reduction in macrophages in the pancreata of *Cxcr2* ^−/−^ mice would have an effect on stellate cell activation. Stellate cell activation, assessed by alpha‐smooth muscle actin (αSMA) staining, was indeed decreased in *Cxcr2* ^−/−^ mice compared with wild‐type (Figure 6D). In this model of chronic pancreatitis, we found that fibrotic tissue and concurrent atrophy accumulated over time (data not shown). At the 9‐month time‐point, picrosirius red staining demonstrated significant pancreatic fibrosis in wild‐type mice (Figure 6E). In some wild‐type mice, however, there was complete atrophy of the gland and very little fibrosis remaining. Thus, when we quantified fibrosis, we could not detect a significant difference between wild‐type and *Cxcr2* ^−/−^ mice (Figure 6F). Taken together, our data show that loss of CXCR2 reduces the number of infiltrating innate immune cells in chronic pancreatitis, thus protecting the pancreatic parenchyma from destruction by neutrophils and macrophages.

### Pharmacological CXCR2 inhibition replicates the protective effects of Cxcr2 deletion

**Figure 7:**
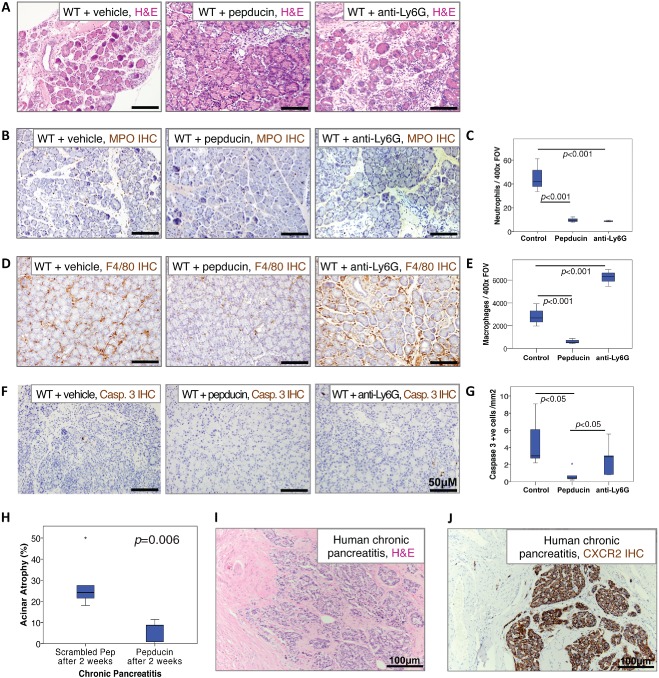
CXCR2 inhibition, but not neutrophil ablation, protects the pancreas from damage associated with chronic pancreatitis. (A) H&E staining of pancreata from vehicle‐treated (left), CXCR2 pepducin‐treated (middle), and anti‐Ly6G‐treated (right) wild‐type mice following 6 weeks of caerulein‐induced chronic pancreatitis. (B) Immunohistochemistry for MPO to detect neutrophils in pancreata from vehicle‐treated (left), CXCR2 pepducin‐treated (middle), and anti‐Ly6G‐treated (right) wild‐type mice following 6 weeks of caerulein‐induced chronic pancreatitis. (C) Boxplot showing quantification of MPO^+^ neutrophils compared between treatment groups. Control versus CXCR2 pepducin, p < 0.001. Control versus anti‐Ly6G, p < 0.001, Mann–Whitney, n = 5 mice in each group. (D) Immunohistochemistry for F4/80 to detect macrophages in pancreata from vehicle‐treated (left), CXCR2 pepducin‐treated (middle), and anti‐Ly6G‐treated (right) wild‐type mice following 6 weeks of caerulein‐induced chronic pancreatitis. (E) Boxplot showing quantification of F4/80^+^ macrophages compared between treatment groups. Control versus CXCR2 pepducin, p < 0.001. Control versus anti‐Ly6G, p < 0.001, Mann–Whitney, n = 5 mice in each group. (F) Immunohistochemistry for cleaved caspase 3 to detect apoptotic cells in pancreata from vehicle‐treated (left), CXCR2 pepducin‐treated (middle), and anti‐Ly6G‐treated (right) wild‐type mice following 6 weeks of caerulein‐induced chronic pancreatitis. (G) Boxplot showing quantification of cleaved caspase 3 compared between treatment groups. Control versus CXCR2 pepducin, p < 0.05. CXCR2 pepducin versus anti‐Ly6G, p < 0.05, Mann–Whitney, n = 5 mice in each group. (H) Boxplot showing quantification of acinar damage in pancreata from wild‐type mice treated with vehicle or CXCR2 pepducin 2 weeks after starting 6 weeks of caerulein‐induced chronic pancreatitis. Control versus CXCR2 pepducin, p < 0.01. Mann–Whitney, n = 5 mice in each group. (I) H&E staining of a section of pancreas resected from a patient with chronic pancreatitis. (J) Immunohistochemistry for CXCR2 in the same patient, showing that dying acinar cells are heavily infiltrated with CXCR2‐positive cells.

Next, we tested the therapeutic potential of CXCR2 inhibition in the prevention and treatment of chronic pancreatitis. We induced chronic pancreatic inflammation in wild‐type mice over the course of 6 weeks, as before (Figure 5), treating them throughout with CXCR2‐inhibiting pepducin, neutrophil‐depleting anti‐Ly6G antibody, or vehicle. As before, in the control group, we observed significant damage to the pancreatic parenchyma and substantial leukocyte infiltration (Figures 7A–7E). Treatment with anti‐Ly6G antibody to deplete neutrophils failed to prevent damage to the pancreas, or modify cell death, as detected by staining for cleaved caspase 3 (Figures 7F and 7G). Remarkably, however, even after 6 weeks of recurrent inflammation, the pancreata of CXCR2 pepducin‐treated mice were significantly protected compared with controls, and far fewer apoptotic cells were present (Figure 7G). Pepducin treatment also resulted in reduced infiltration of the pancreas by both MPO^+^ neutrophils and F4/80^+^ monocytes (Figures 7B–7E). Anti‐Ly6G antibody‐treated mice also displayed fewer neutrophils in their pancreata (Figures 7B and 7C). However, the macrophage number was increased, even when compared with control‐treated mice (Figures 7D and 7E). Neutrophil ablation alone is therefore insufficient to provide the same pancreatic protection as CXCR2 inhibition, with monocytes infiltrating the pancreas, causing pancreatic damage. These data suggest that CXCR2 inhibition does indeed protect the pancreas from chronic pancreatic inflammation, first through inhibition of neutrophil chemotaxis and second through neutrophil‐independent processes, potentially disruption of the coordination of the innate immune response, and interference with monocyte chemotaxis. Encouragingly, even when treatment started 2 weeks after induction of pancreatitis, pepducin could ‘rescue’ the pancreas from damage (Figure 7H). When we examined expression of CXCR2 in tissue from patients with chronic pancreatitis, we found that while a substantial proportion of the pancreas had been replaced by fibrotic tissue (Figure 7I), dying acini were heavily infiltrated with CXCR2‐positive cells (Figure 7J). Given that CXCR2 inhibitors are now available in the clinic 40, 41, we believe that these data provide support for future CXCR2‐based therapeutic intervention.

## Discussion

We have shown here that there is excellent rationale for the use of CXCR2 inhibitors in pancreatic inflammation. Many of the known molecular processes characterized in acute pancreatitis converge on CXCR2 signalling and stimulate CXC chemokine release. Our data demonstrate an important role for CXCR2 in mediating the innate immune cell response characteristic of pancreatic inflammation. By extension of our studies from acute pancreatitis, through recurrent acute inflammation, to the changes of chronic pancreatitis, we have demonstrated the importance of CXCR2‐mediated immune cell infiltration to the propagation of all stages of pancreatic inflammation. We have confirmed the importance of neutrophils in the pathogenesis of acute pancreatitis both in its early stages and 24 h after initiation 14. CXCR2 deficiency, resulting from genetic knockout or pharmacological inhibition, prevented chemotaxis of these cells to the pancreas, an effect equivalent to neutrophil ablation. We have also shown, through conditional deletion of *Cxcr2* in myeloid cells, that CXCR2 signalling in myeloid cells is vital for the infiltration of these cells to the pancreas.

Furthermore, we have shown that CXCR2 signalling links the progression of persistent acute inflammation to changes associated with chronic pancreatitis. CXCR2 deficiency, both genetic and pharmacologically mediated, protected against innate immune cell infiltration and the damage incurred following repeated pancreatic inflammation. Prolongation of recurrent acute inflammation studies to 9 months generates a model that incorporates inflammation with resultant atrophy and fibrosis and is pathologically more akin to chronic pancreatitis. In this context, CXCR2 deficiency once again protected against innate immune cell infiltration, with reductions in both neutrophil and macrophage populations evident. Furthermore, CXCR2 deletion protected against atrophic changes within the pancreas when chronic pancreatitis was induced. Human chronic pancreatitis specimens displayed significant fibrosis and acinar atrophy. CXCR2 staining was very high in areas of acinar atrophy and exclusively expressed within immune cells. Thus, our data support the belief that chronic pancreatitis arises from recurrent pancreatic inflammation.

Other authors have observed similar effects of inhibiting neutrophils in the context of acute pancreatitis 42. Indeed, neutrophils have the potential to activate acinar cell trypsinogen 13, and neutrophil‐produced reactive oxygen species are likely to have a key role in this process 14. Recently, it has been shown that trypsinogen activation is important only in early acute pancreatitis, as trypsinogen‐null mice demonstrated similar levels of NF‐κB expression to controls in the setting of AP 2. NF‐κB activation, and not trypsinogen activation, is now considered the important step in maintaining the immune response in acute pancreatitis. We have shown that CXCR2 deficiency can interfere with NF‐κB‐dependent progression of acute pancreatitis.

Interestingly, in acute pancreatitis, prevention of neutrophil chemotaxis was sufficient to prevent subsequent monocyte infiltration and thereby protect the pancreas from the necrotizing effects of these cells. However, we have shown that in the context of recurrent acute inflammation, CXCR2 has effects over and above inhibiting neutrophils alone, as CXCR2 inhibition prevented monocyte infiltration that in control and neutrophil ablated models led to tissue damage and necrosis. Thus, CXCR2 may be expressed by macrophages in chronic inflammatory settings, as has been shown previously in atherosclerosis 43, or perhaps in the absence of neutrophils, stimulus for monocyte infiltration comes from an innate mechanism stimulated by neutrophils 44. Indeed, we cannot rule out the possibility that upon neutrophil infiltration, those few tissue‐resident macrophages present play a role in mediating the chronic effects. CXCR2 knockout protected against atrophic changes within the pancreas when chronic pancreatitis was induced. Thus, CXCR2 deficiency protects pancreatic parenchyma in a fashion dependent on neutrophil and subsequent monocyte infiltration, although interestingly, *Cxcr2* deletion does appear to prevent stellate cell activation.

There is some degree of fibrosis in *Cxcr2* ^−/−^ pancreata, which may be explained in different ways. *Cxcr2* loss fails to block infiltration of all monocytes to the pancreas and those that do reach the pancreas may be sufficiently able to activate stellate cells. Given the very small numbers of macrophages within the pancreas, however, this seems unlikely. Stellate cells may also be activated by cytokines released in an autocrine fashion in response to damage 45, or become activated in response to oxidative stress experienced by the pancreas following repeated caerulein injections 46.

Clinically relevant inhibitors of CXCR2 are now available for trial in patients 40, 41. We demonstrated the ability of CXCR2 inhibition to interfere in the necrotic process of acute pancreatitis, suggesting the potential for therapeutic trial in both acute and chronic pancreatitis. Although further work is required to assess redundancy between CXCR1 and CXCR2 in humans, dual inhibitors such as reparixin are also available for use in the clinic 47. Many patients are at risk of recurrent pancreatic inflammation, with no clear aetiology being diagnosed in 10–30% of cases of acute recurrent pancreatitis 48. The clear association between CXCR2‐positive cells and acinar collapse observed in human chronic pancreatitis suggests that CXCR2 may be a powerful therapeutic target in these patients.

## Author contribution statement

The authors contributed in the following way: CWS: study design, data acquisition, data analysis, and drafting of the manuscript; SAK: data acquisition; MF: data analysis; LR: data acquisition; RJP: data analysis; JDGL: data analysis; CN: data acquisition; TRJE: study supervision; CRC: study supervision and critical reading of the manuscript; RJBN: study concept and design, data analysis, and drafting of the manuscript; OJS: study concept and design, study supervision, data analysis, and drafting of the manuscript; JPM: study concept and design, study supervision, data acquisition, data analysis, and drafting of the manuscript. All authors read and agreed on the final manuscript.

**SUPPORTING INFORMATION ON THE INTERNET**

The following supporting information may be found in the online version of this article:

**Supplementary materials and methods.**

**Figure S1.** Immune cell infiltration in *Cxcr2* WT mice compared with *Cxcr2^−/−^* mice.

**Figure S2.** Very few immune cells infiltrate the pancreas in untreated mice.

**Figure S3.** Full blood counts (FBCs) performed on blood from *Cxcr2* WT and *Cxcr2^−/−^* mice, and under control and acute inflammatory conditions.

**Figure S4.** Pancreas‐specific CXCR2 deletion does not protect from pancreatitic inflammation.

**Figure S5.** Full blood counts (FBCs) performed on blood from *Cxcr2* WT and *Cxcr2^−/−^* mice, and under control and chronic inflammatory conditions.

**Figure S6.** Activation of NF‐κB signalling in both acute and chronic pancreatitis.

## Supporting information


**AppendixS1.** Supplementary materials and methods

**Immune cell infiltration in Cxcr2 WT mice compared with Cxcr2^−/−^ mice.** A) Immunohistochemistry for F4/80 detecting macrophages in the pancreas 24 hours post‐induction of acute pancreatitis in wild‐type and Cxcr2^−/−^ mice. B) Immunohistochemistry for CD3 detecting T cells in the pancreas of Cxcr2 WT and Cxcr2^−/−^ mice 24 hours post‐induction of acute pancreatitis.

**Very few immune cells infiltrate the pancreas in untreated mice.** Flow cytometric analysis for neutrophils (CD11b^+^ Ly6G^+^) and macrophages (CD11b^+^ F4/80^+^) in cells isolated from the pancreata of untreated wild‐type mice.

**Full blood counts (FBCs) performed on blood from Cxcr2 WT and Cxcr2^−/−^ mice, and under control and acute inflammatory conditions.** A‐D) Numbers of circulating A) neutrophils, B) monocytes, C) lymphocytes, and D) white blood cells (WBCs), in Cxcr2 WT and Cxcr2^−/−^ mice that were untreated (control), or sacrificed 24 h following acute pancreatitis induction (AP). D‐F) Number of circulating D) neutrophils, E) monocytes and F) lymphocytes, are shown expressed as a percentage of WBCs in Cxcr2 WT and Cxcr2^−/−^ mice (n ≥3, * Mann‐Whitney P < 0.05).

**Pancreas‐specific CXCR2 deletion does not protect from pancreatitic inflammation.** A) H&E staining of pancreata harvested from (A) wild‐type and (B) Pdx1‐Cre, Cxcr2^fl/fl^ mice following 6 weeks of pancreatic inflammation. (C) Boxplot showing quantification of neutrophils within the pancreas of wild‐type and Pdx1‐Cre, Cxcr2^fl/fl^ mice following 6 weeks of pancreatic inflammation, n = 5 mice.

**Full blood counts (FBCs) performed on blood from Cxcr2 WT and Cxcr2^−/−^ mice, and under control and chronic inflammatory conditions.** A‐D) Numbers of circulating A) neutrophils, B) monocytes, C) lymphocytes, and D) white blood cells (WBCs), in Cxcr2 WT and Cxcr2^−/−^ mice that were untreated (control), or sacrificed 6 weeks after induction of chronic inflammation (CP). D‐F) Number of circulating D) neutrophils, E) monocytes and F) lymphocytes, are shown expressed as a percentage of WBCs in Cxcr2 WT and Cxcr2^−/−^ mice (n ≥3, * Mann‐Whitney P < 0.05).

**Activation of NF‐κB signalling in both acute and chronic pancreatitis.** A‐B) Immunohistochemistry for NF‐κB‐p65 in the pancreas of Cxcr2 WT and Cxcr2^−/−^ mice, A) 24 hours post‐induction of acute pancreatitis or B) 6 weeks after induction of chronic pancreatitis. Note nuclear staining.
