# Supplementary material for: CXCR2 inhibition suppresses acute and chronic pancreatic inflammation
Source: J Pathol. 2015 Jun 4;237(1):85–97. doi: 10.1002/path.4555 (PMC4833178; doi:10.1002/path.4555)
Supplement: Supplementary file 1 — AppendixS1. Supplementary materials and methods [file PATH-237-85-s001.doc]

**SUPPORTING INFORMATION**

**Supplementary materials and methods**

**Treatment Studies**

For neutrophil depletion studies 20mg/kg of either anti-Ly6G monoclonal antibody (1A8, BioXcell) or isotype control (2A3 rat anti-mouse IgG, BioXcell) was injected intraperitoneally (3 times per week via for the duration of study. For CXCR2 inhibition mice were injected subcutaneously with X1/2-ipal-i3 pepducin (RTLFKAHMGQKHR, palmitoyl N-terminal, amidation C-terminal; Genscript) or control (TRFLAKMHQGHKR, palmitoyl N-terminal, amidation C-terminal; Genscript) at a dose of 2.5mg/kg on day one, followed by 1mg/kg daily for the duration of the study.

**Immunohistochemistry**

Animals were sacrificed at time-points and pancreata harvested and fixed in either 10% neutral buffered formalin or methacarn (methanol: chloroform: acetic acid; 4:2:1). The tissue was paraffin-embedded and 5μm sections used for haematoxylin and eosin staining, and immunohistochemistry.

The following antibodies were used: anti-Myeloperoxidase (MPO) (Dako A0398) 1:200; anti-F4/80 clone A3-1 (Abcam ab6640) 1:400; anti-cleaved caspase 3 clone SP175 (Cell Signaling 9661) 1:50; anti-Ki67 clone SP6 (Thermo RM-9106) 1:200; anti-mouse CXCR2 clone 242216 (R&D systems MAB2164-100) 1:500; anti-human CXCR2 clone 19 (Invitrogen AHR1532X) 1:200; anti-alpha smooth muscle actin (αSMA) clone 1A4 (Sigma-Aldrich A2547) 1:50000.

Cells were scored at x200 or x400 field of view (FOV). At least 3 mice per group were assessed. A minimum of 20 FOV or the entire pancreas was examined. Data were analysed using SPSS (IBM) statistical software and non-parametric Mann-Whitney analysis used to compare groups. To quantify stains that cannot be quantified by cell number, we took pictures of more than 30 fields of view at x40 magnification in one sitting to account for light and picture variability. We used Adobe Photoshop 5.1 to pixel count the colour of positive staining as a surrogate for quantity. Boxplots throughout the results display the mean value and the interquartile range, while the whiskers demarcate the extremes of data.

**Flow Cytometric Analysis**

The single cells were isolated from pancreas by cold trypsin-EDTA digestion. Briefly, tissue fragments were soaked in 0.25% trypsin-EDTA for 1h or 24 h at 4°C. After the incubation, tissue fragments were further digested in 10ug/ml DNase at 37°C for 10 minutes. The isolated single cells were stained with 1:200 diluted anti-mouse CD11b, F4/80, Ly-6G, CD3 or B220 (all eBioscience) for 20 minutes on ice, washed and suspended in FACS-buffer (PBS-2% FBS). Cells were analyzed by flow cytometer.
