# Supplementary figures and images for: CXCR2 inhibition suppresses acute and chronic pancreatic inflammation
Source: J Pathol. 2015 Jun 4;237(1):85–97. doi: 10.1002/path.4555 (PMC4833178; doi:10.1002/path.4555)

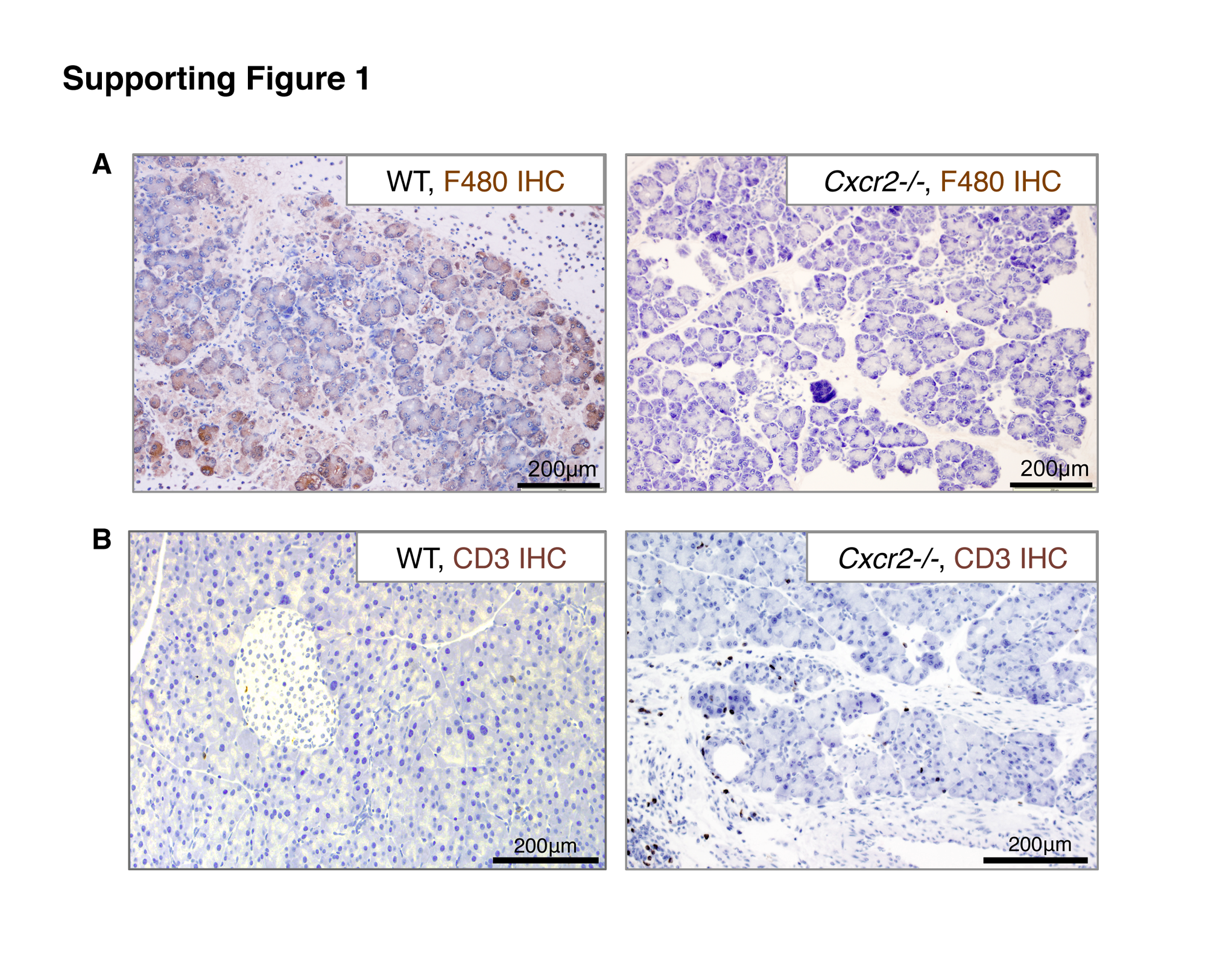

Supplement: Supplementary file 2 — Immune cell infiltration in Cxcr2 WT mice compared with Cxcr2−/− mice. A) Immunohistochemistry for F4/80 detecting macrophages in the pancreas 24 hours post‐induction of acute pancreatitis in wild‐type and Cxcr2−/− mice. B) Immunohistochemistry for CD3 detecting T cells in the pancreas of Cxcr2 WT and Cxcr2−/− mice 24 hours post‐induction of acute pancreatitis. [file PATH-237-85-s002.tif]

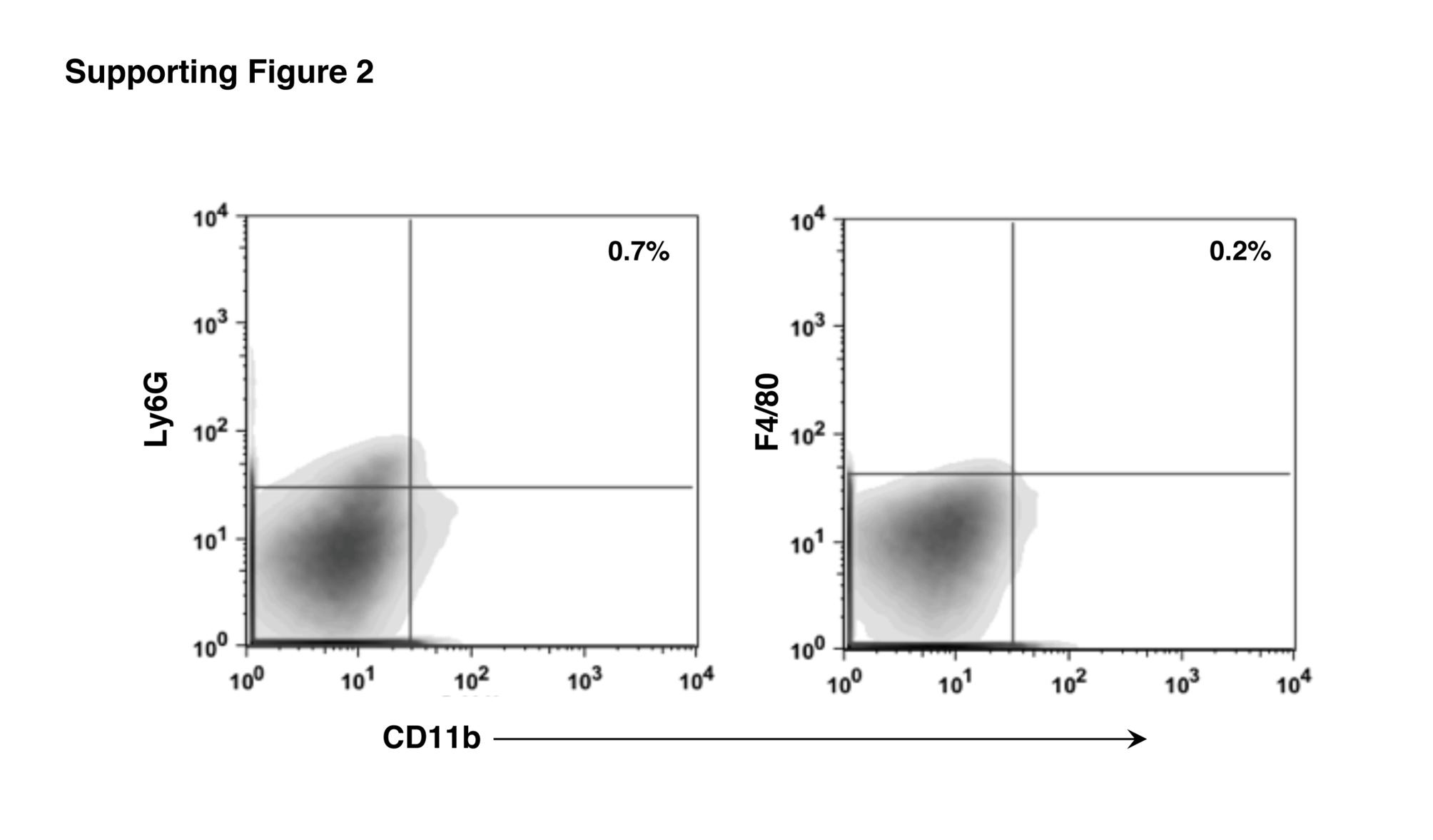

Supplement: Supplementary file 3 — Very few immune cells infiltrate the pancreas in untreated mice. Flow cytometric analysis for neutrophils (CD11b+ Ly6G+) and macrophages (CD11b+ F4/80+) in cells isolated from the pancreata of untreated wild‐type mice. [file PATH-237-85-s003.tif]

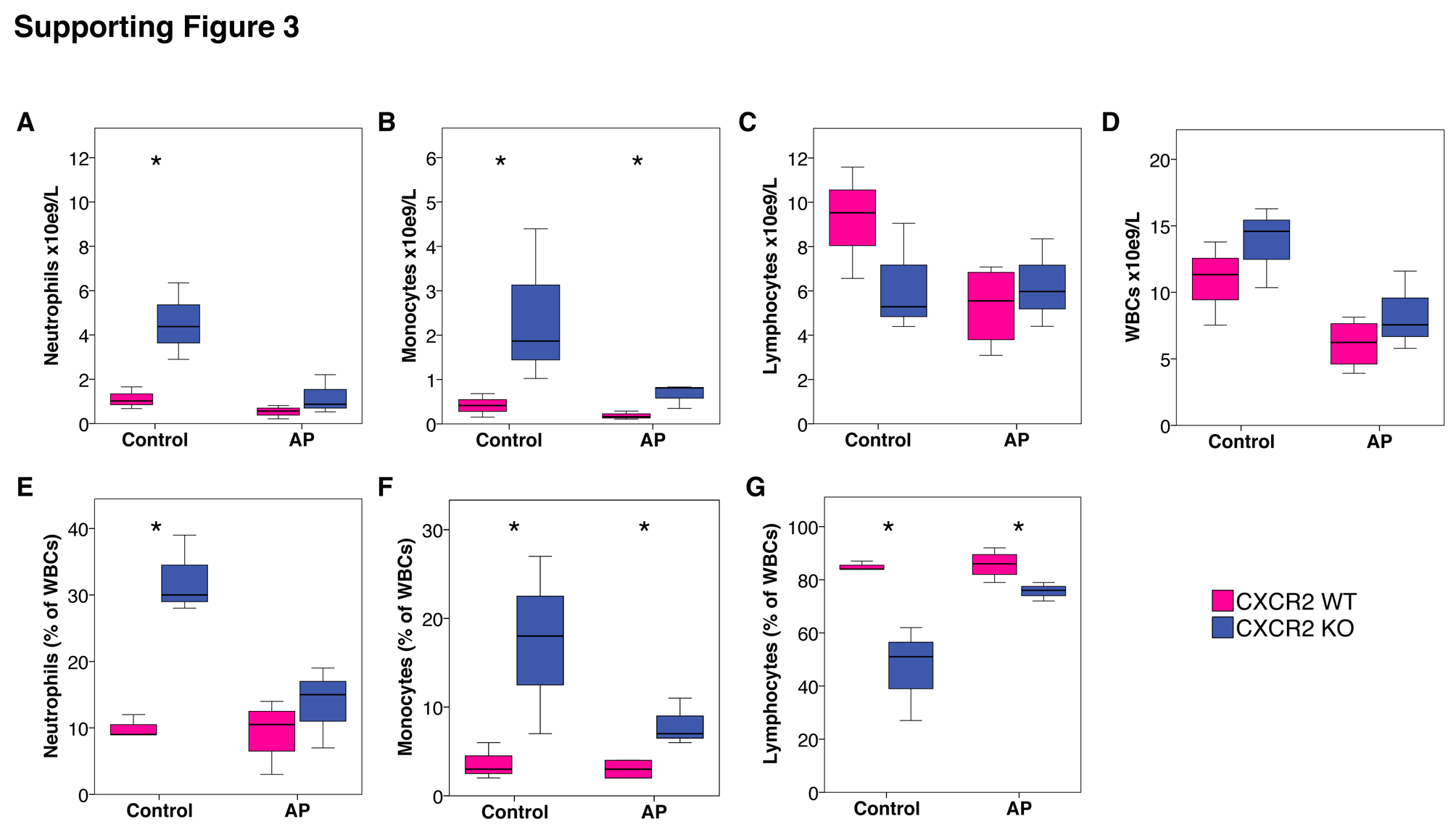

Supplement: Supplementary file 4 — Full blood counts (FBCs) performed on blood from Cxcr2 WT and Cxcr2−/− mice, and under control and acute inflammatory conditions. A‐D) Numbers of circulating A) neutrophils, B) monocytes, C) lymphocytes, and D) white blood cells (WBCs), in Cxcr2 WT and Cxcr2−/− mice that were untreated (control), or sacrificed 24 h following acute pancreatitis induction (AP). D‐F) Number of circulating D) neutrophils, E) monocytes and F) lymphocytes, are shown expressed as a percentage of WBCs in Cxcr2 WT and Cxcr2−/− mice (n ≥3, * Mann‐Whitney P < 0.05). [file PATH-237-85-s004.tif]

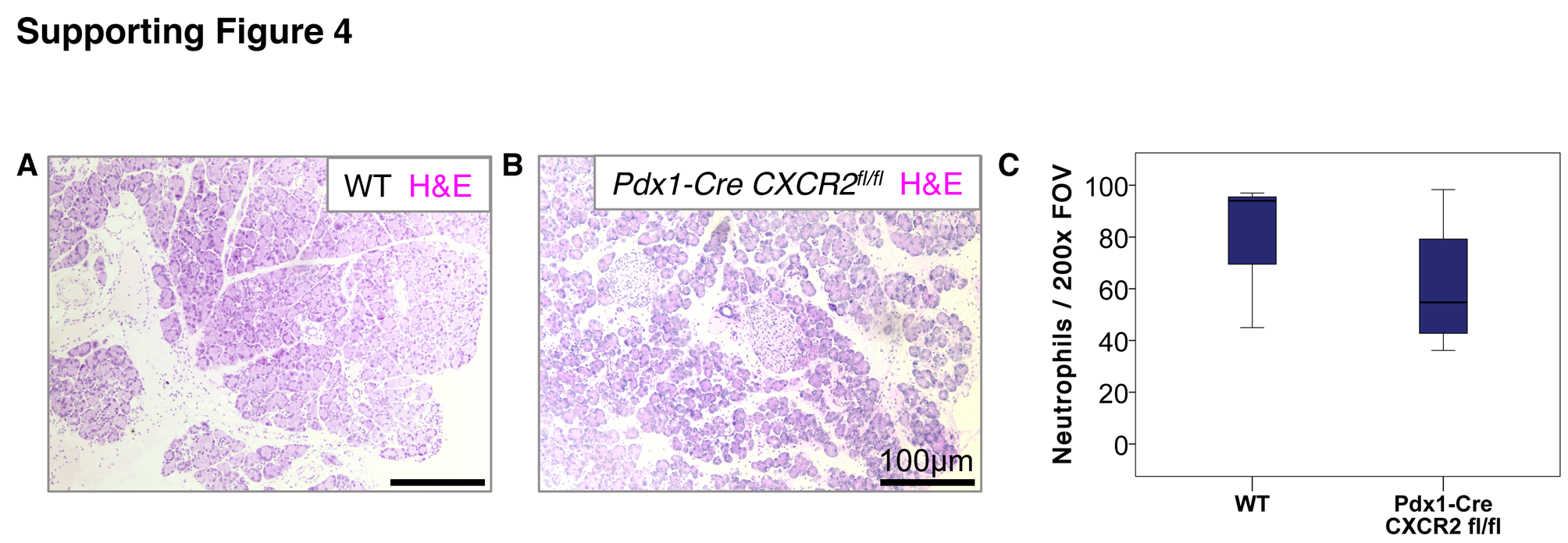

Supplement: Supplementary file 5 — Pancreas‐specific CXCR2 deletion does not protect from pancreatitic inflammation. A) H&E staining of pancreata harvested from (A) wild‐type and (B) Pdx1‐Cre, Cxcr2fl/fl mice following 6 weeks of pancreatic inflammation. (C) Boxplot showing quantification of neutrophils within the pancreas of wild‐type and Pdx1‐Cre, Cxcr2fl/fl mice following 6 weeks of pancreatic inflammation, n = 5 mice. [file PATH-237-85-s005.tif]

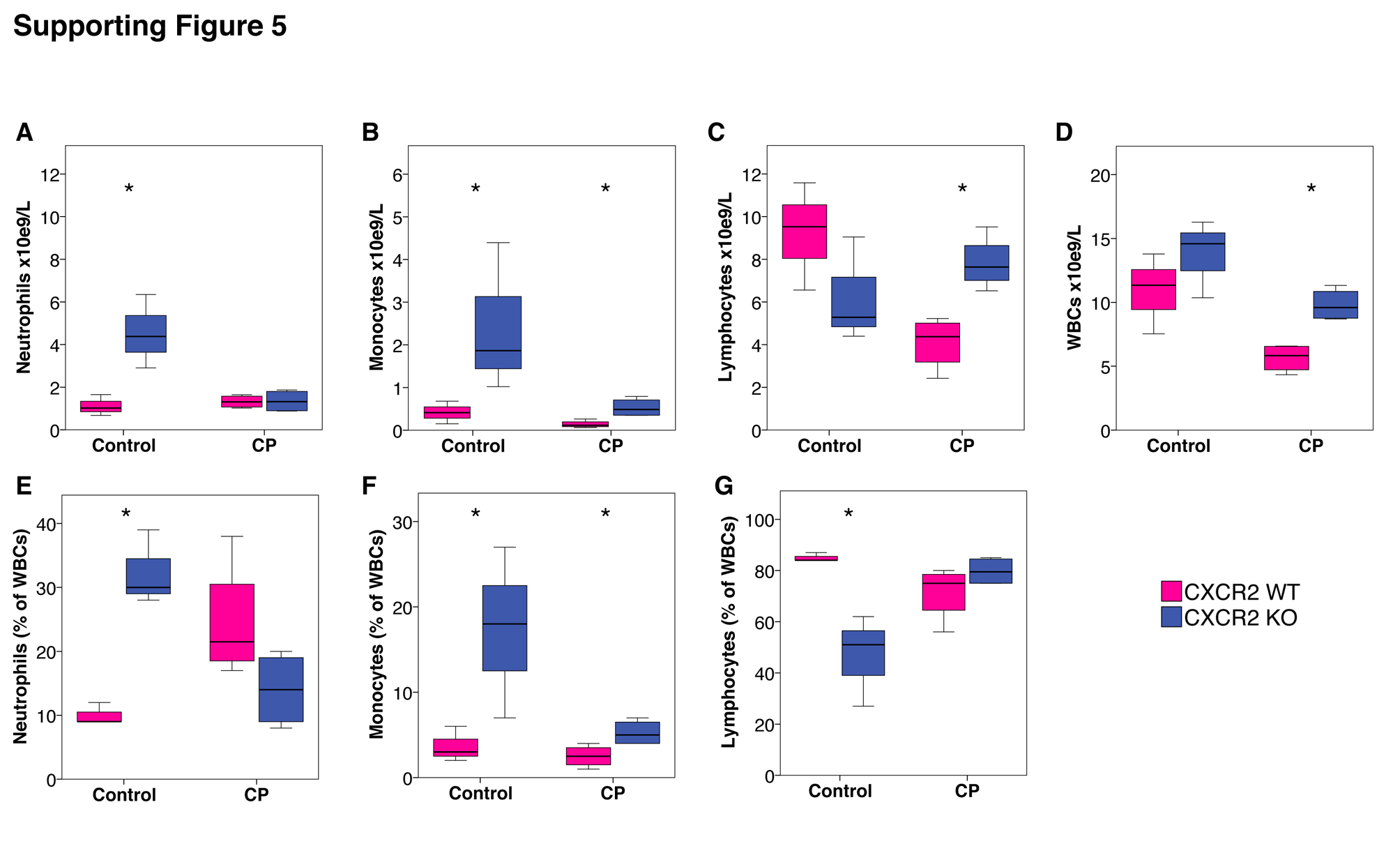

Supplement: Supplementary file 6 — Full blood counts (FBCs) performed on blood from Cxcr2 WT and Cxcr2−/− mice, and under control and chronic inflammatory conditions. A‐D) Numbers of circulating A) neutrophils, B) monocytes, C) lymphocytes, and D) white blood cells (WBCs), in Cxcr2 WT and Cxcr2−/− mice that were untreated (control), or sacrificed 6 weeks after induction of chronic inflammation (CP). D‐F) Number of circulating D) neutrophils, E) monocytes and F) lymphocytes, are shown expressed as a percentage of WBCs in Cxcr2 WT and Cxcr2−/− mice (n ≥3, * Mann‐Whitney P < 0.05). [file PATH-237-85-s006.tif]

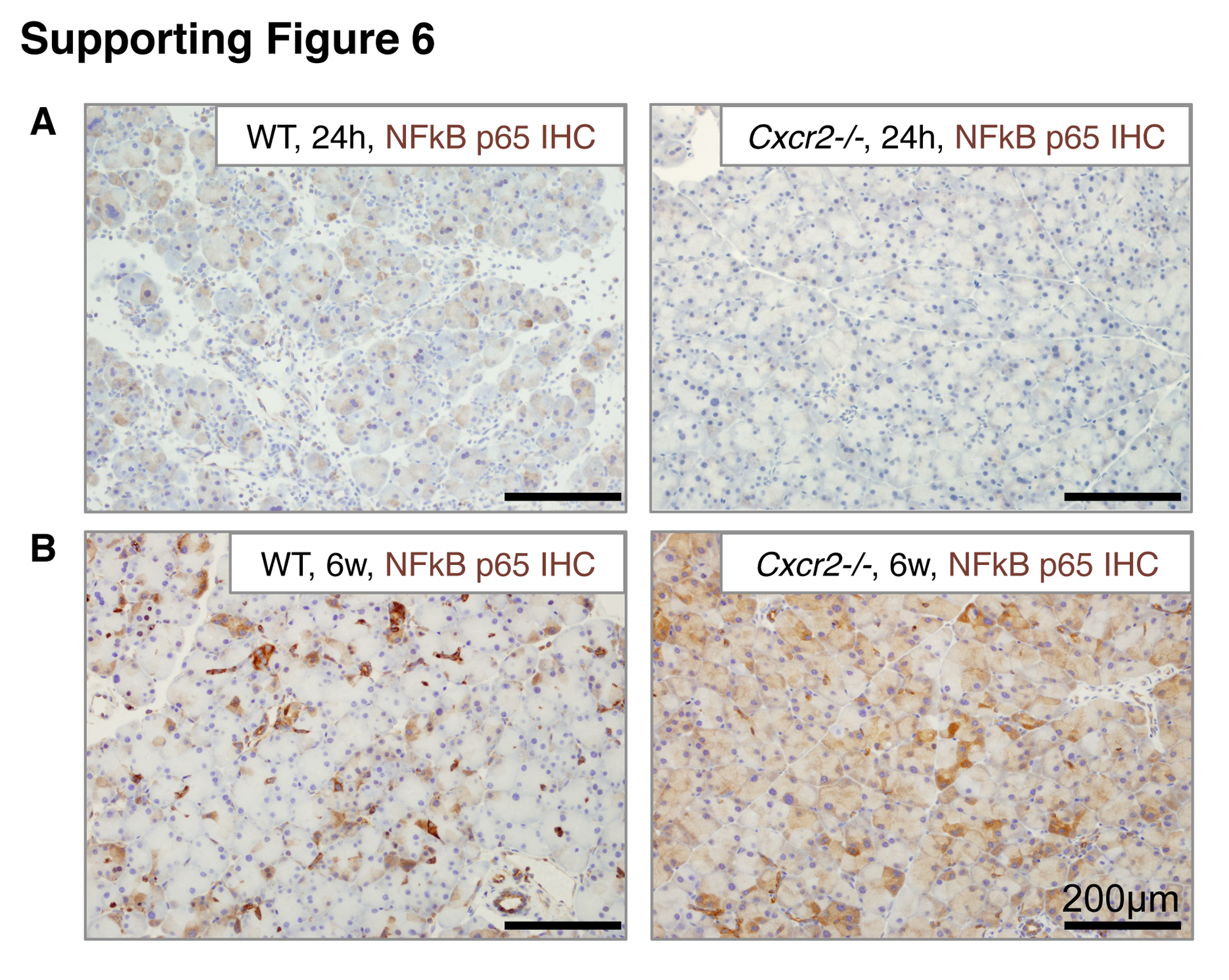

Supplement: Supplementary file 7 — Activation of NF‐κB signalling in both acute and chronic pancreatitis. A‐B) Immunohistochemistry for NF‐κB‐p65 in the pancreas of Cxcr2 WT and Cxcr2−/− mice, A) 24 hours post‐induction of acute pancreatitis or B) 6 weeks after induction of chronic pancreatitis. Note nuclear staining. [file PATH-237-85-s007.tif]
